# High extinction risk in large foraminifera during past and future mass extinctions

**DOI:** 10.1126/sciadv.adj8223

**Published:** 2024-08-07

**Authors:** Yan Feng, Haijun Song, Hanchen Song, Yuyang Wu, Xing Li, Li Tian, Shuaishuai Dong, Yanli Lei, Matthew E. Clapham

**Affiliations:** ^1^ State Key Laboratory of Biogeology and Environmental Geology, School of Earth Sciences, China University of Geosciences, Wuhan 430074, China.; ^2^ Department of Marine Organism Taxonomy & Phylogeny, Institute of Oceanology, Chinese Academy of Sciences, Qingdao 266071, China.; ^3^ College of Marine Science and Fisheries, Jiangsu Ocean University, Lianyungang 222005, China.; ^4^ Department of Earth and Planetary Sciences, University of California, Santa Cruz, Santa Cruz, CA 95064, USA.

## Abstract

There is a strong relationship between metazoan body size and extinction risk. However, the size selectivity and underlying mechanisms in foraminifera, a common marine protozoa, remain controversial. Here, we found that foraminifera exhibit size-dependent extinction selectivity, favoring larger groups (>7.4 log_10_ cubic micrometer) over smaller ones. Foraminifera showed significant size selectivity in the Guadalupian-Lopingian, Permian-Triassic, and Cretaceous-Paleogene extinctions where the proportion of large genera exceeded 50%. Conversely, in extinctions where the proportion of large genera was <45%, foraminifera displayed no selectivity. As most of these extinctions coincided with oceanic anoxic events, we conducted simulations to assess the effects of ocean deoxygenation on foraminifera. Our results indicate that under suboxic conditions, oxygen fails to diffuse into the cell center of large foraminifera. Consequently, we propose a hypothesis to explain size distribution–related selectivity and Lilliput effect in animals relying on diffusion for oxygen during past and future ocean deoxygenation, i.e., oxygen diffusion distance in body.

## Teaser

Large foraminifera are more vulnerable to extinction during ocean deoxygenation.

## INTRODUCTION

Biodiversity on modern Earth is being lost at an unprecedented rate, with extinction rates far exceeding the background extinction rates (*1*). A number of extinction events occurred during the Phanerozoic, with the best known “Big Five” mass extinctions occurring in the end-Ordovician (445.2 Ma), Frasnian-Famennian (F-F, 372.2 Ma), Permian-Triassic (P-T, 251.9 Ma), Triassic-Jurassic (T-J, 201.3 Ma), and Cretaceous-Paleogene (K-Pg, 66.0 Ma) (*2*, *3*). Therefore, some scientists have suggested that Earth is already experiencing the “sixth mass extinction” (*4*). Both the fossil record and modern data suggest that some species are more vulnerable to extinction risk than others (*5*).

The selectivity of extinction risk is frequently related to many factors, including abundance (*6*), geographic range (*7*), species richness per genus (*8*), and body size (*9*). One of the longest-standing controversies is the relationship between body size and extinction risk (*9*). The fossil record suggests that small-bodied mollusks and fishes had higher extinction rates during the P-T, T-J, and K-Pg mass extinctions (*5*), but some large fishes and tetrapods preferentially went extinct during the end-Devonian mass extinction (*10*). Large-bodied anurans also declined after the K-Pg mass extinction (*11*). In modern studies, large-bodied mollusks and fishes are often at higher risk of extinction (*12*).

Compared to these well-known research interests in macroscopic animals, microscopic protozoa are less understood in terms of size selectivity during mass extinctions. Benthic foraminifera are abundant, diverse, and widely distributed in the oceans, with a long evolutionary history that began more than 500 million years ago (*13*). Modern foraminifera in many regions have been severely affected by human activities as well as environmental and climatic changes (*14*). The fossil record indicates size selectivity among benthic foraminifera during the P-T mass extinction event, with larger foraminifera exhibiting a higher susceptibility to extinction (*15*–*18*). Similarly, during the K-Pg mass extinction, larger planktonic foraminifera exhibited a trend of preferential extinction (*19*). In contrast, fusulinoidean foraminifers did not exhibit size-selective extinction during the Guadalupian-Lopingian (G-L) extinction event (*20*). However, the extinction risk for foraminifera during other mass extinctions, as well as their modern extinction risk, remains unclear. In addition, it is uncertain whether the observed size selectivity in the same foraminiferal group, such as benthic foraminifera, is consistent across different extinction events. Therefore, foraminifera represent a suitable group for investigating the quantitative relationship between body size and extinction risk. In this study, we used a new foraminiferal size dataset to investigate the potential size selectivity of benthic foraminifera in past mass extinctions and in the modern ocean.

## RESULTS

**Fig. 1.**
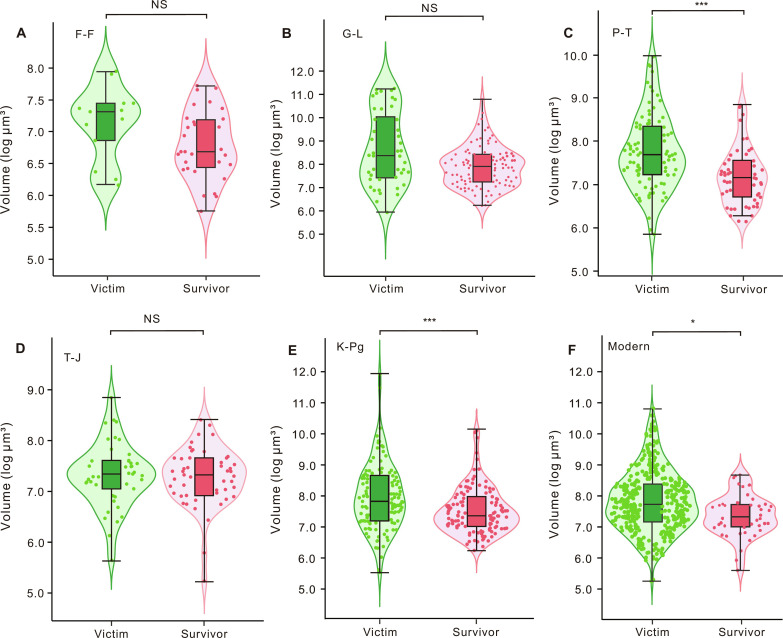
Violin and box plots of body size (log_10_ μm^3^) of extinct and surviving foraminiferal genera during mass extinctions and modern times. (**A**) F-F mass extinction. (**B**) G-L extinction. (**C**) P-T mass extinction. (**D**) T-J mass extinction. (**E**) K-Pg mass extinction. (**F**) Modern times. The terms “victim” and “survivor” in modern times represent foraminiferal taxa at risk of extinction and those not at risk of extinction, respectively. The light green (left) and light red (right) shapes represent the kernel density plots of the distribution of extinct and surviving foraminifera, respectively. Filled circles show the mean value of each foraminiferal genus. Box plots show the median (the black horizontal line inside the box) and interquartile range (upper and lower limits of the box) of the extinct and surviving foraminifera, respectively. The Mann-Whitney *U* test was used to examine differences in body size between victims and survivors. ***P < 0.001; *, 0.01 < *P* < 0.05; NS, *P* > 0.05.

Violin and box plots show the body size distribution of foraminiferal victims and survivors in the F-F, G-L, P-T, T-J, and K-Pg extinctions and modern extinction risk (Fig. 1). For the P-T (*P* < 0.001) and K-Pg (*P* < 0.001) mass extinctions and modern times (*P* = 0.015), there were significant differences in body size between victims and survivors (table S1). Specifically, the median test biovolume of extinct genera (7.69 log_10_ μm^3^) was 3.47 times (10^7.69^/10^7.15^ = 3.47) that of survivors (7.15 log_10_ μm^3^) during the P-T mass extinction. The median sizes of extinct foraminiferal genera in the K-Pg mass extinction and modern era were 3.89 times and 2.51 times larger than those of survivors, respectively. The median sizes of victims in the F-F (*P* = 0.627) and G-L (*P* = 0.101) extinction events were larger than those of survivors, but the differences were not significant. During the T-J mass extinction event, the median sizes for extinct and surviving foraminifera were similar.

**Fig. 2.**
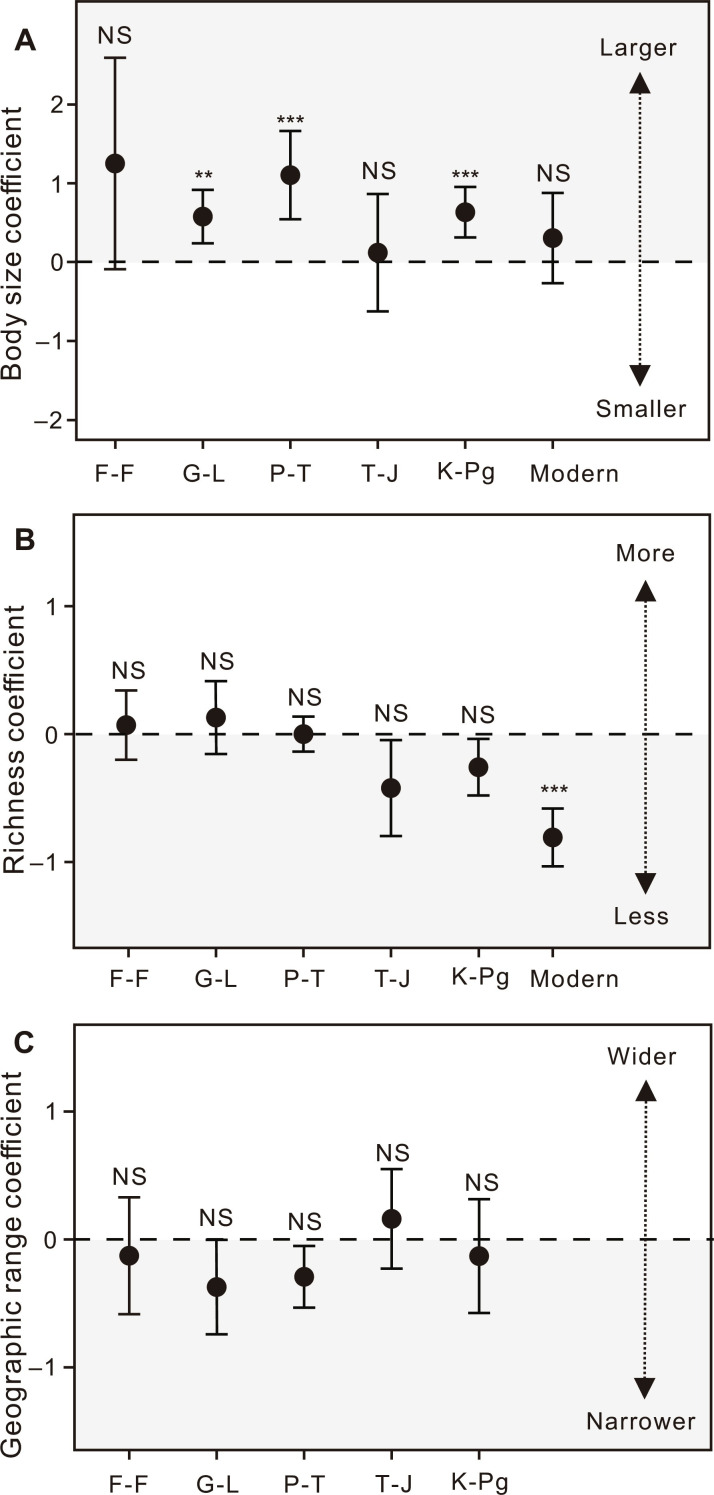
Regression coefficients from multiple logistic regression analyses of extinction as a function of various factors. (**A**) Body size, (**B**) Species richness, and (**C**) Geographic range. Error bars represent 95% confidence intervals for the estimated regression coefficients. The gray shading indicates that our results are mostly located on this side, i.e., the large body size, low species richness, and narrow geographic distribution of foraminiferal genera are preferentially extinct, with the same abbreviations as above. ***P < 0.001; **P < 0.01; NS, *P* > 0.05.

We also used logistic regression analysis to explore the relationships among three key metrics—body size, species richness, and geographic range—and extinction risk. Notably, size selectivity was significant in the G-L (*P* = 0.007), P-T (*P* < 0.001), and K-Pg (*P* < 0.001) extinctions, i.e., larger foraminifera led to greater extinction risk, whereas it was not significant (*P* > 0.05) in the F-F and T-J mass extinctions (Fig. 2A). Size selectivity was not significant in modern extinction risk (Fig. 2A), but there was a significant size difference between genera at extinction risk and those not endangered (Fig. 1). This is because we took into account the possible effects of species richness in our logistic regression analyses.

Conversely, we observed no significant selectivity with respect to species richness per genus during extinction events (*P* > 0.05; table S2 and Fig. 2B), which indicates that the relationship between extinction risk and species richness in foraminifera is unclear. When assessing modern extinction risk, we observed a significant negative correlation, suggesting that genera with lower species richness are at higher risk of extinction. Figure 2C shows the relationship between geographic range and extinction risk during extinctions. The relationship between geographic range and extinction risk was not significant (*P* > 0.05; table S2), implying that variations in geographic range of foraminifera did not predict their extinction risk during these events. Logistic regression analyses were not performed between the geographic range and extinction risk in the modern era, as the extinction risk of contemporary foraminifera covaries with their geographic range.

## DISCUSSION

### Size selectivity in past mass extinctions and modern extinction risk

Multiple logistic regression analysis showed that body size was the most consistent predictor of extinction selectivity compared to geographic range and species richness. The coefficients for body size are consistently greater than 0 (Fig. 2), suggesting a tendency for large-bodied foraminifera to face preferential extinction. Size selectivity was significant during the G-L (*P* = 0.007), P-T (*P* < 0.001), and K-Pg (*P* < 0.001) extinctions. Although it was not significant (*P* > 0.05) during the F-F and T-J mass extinctions and in the modern times, a trend toward higher extinction risk of large taxa is evident (Fig. 2A).

Geographic range was not significantly associated with extinction risk (Fig. 2C). During the F-F, G-L, P-T, and K-Pg extinctions, a weak negative association was observed between geographic range and extinction risk (table S2). This finding is similar to previous studies, which indicated that mass extinctions and some second-order extinctions exhibited very weak geographic range selectivity (*6*, *7*). However, for the T-J mass extinction, the relationship between geographic range and extinction risk was not consistent (table S2). We also observed no consistent relationship between species richness of foraminifera and extinction risk during mass extinctions. Species richness per genus was not clearly associated with the extinction risk (*6*, *7*).

Our results suggest that larger foraminifera preferentially went extinct during the G-L, P-T, and K-Pg extinctions (Figs. 1 and 2). However, the extinction selectivity of metazoans is controversial. Payne *et al.* (*5*) documented that small-bodied marine vertebrates and mollusks were preferentially affected during the P-T, T-J, and K-Pg extinctions. Monarrez *et al.* (*21*) suggested that larger trilobites and cephalopods were associated with a higher extinction risk during mass extinctions. These results may indicate that size-based extinction selectivity varied in different taxonomic groups. This may also be due to the different temporal resolutions of the body size data collected. Previous studies applied the size of the largest specimen within the entire range of a genus to every stage (*5*, *21*). In this study, the body size of foraminiferal genera was based on fossil specimens specifically from the stage containing the extinction event.

Intense environmental stress undoubtedly contributes to the preferential extinction of large individuals during mass extinctions and modern extinction risk (fig. S1). The F-F, G-L, P-T, T-J, and K-Pg extinction events were all associated with large igneous provinces corresponding to the Viluy Traps, Emeishan, Siberian, Central Atlantic, and Deccan volcanisms, respectively (*2*). Volcanic eruption–induced climate change, ocean anoxia, and acidification are the most likely driving mechanisms of mass extinctions (*2*, *22*–*24*). In addition to the Viluy Traps, nutrient runoff from the first landings of forest and terrestrial plants in the F-F mass extinction event caused ocean eutrophication and eventually anoxia (*25*). The Chicxulub asteroid impact not only altered the nutrient structure of the ocean, leading to the extinction of large benthic foraminifera due to the loss of their habitats (*26*, *27*). In addition, the “impact winter” may have played an important role in the K-Pg mass extinction, injecting dust, soot, and sulphur aerosols into the atmosphere; partially blocking solar radiation; severely disrupting photosynthesis; and leading to widespread phytoplankton die-offs, thus affecting the entire ecosystem (*28*–*31*). Larger foraminifera are more sensitive to hypoxia (*32*) because they have a relatively small surface area per unit volume, which impairs efficient oxygen uptake and therefore lower rates of oxygen uptake, but their large body requires more oxygen in total (*33*). In addition, large foraminifera tend to have long lifespans, and their reproductive strategies are more akin to K-strategies, making them more susceptible to extinction in catastrophic environments (*27*, *34*).

Industrial and agricultural pollutants and discharge of municipal sewage are important factors that lead to extinction risk to foraminifera (fig. S1). These factors not only bring toxic materials to the oceans but also contribute to marine eutrophication (*35*). This is similar to the environmental stresses in the F-F mass extinction event, when eutrophication of the ocean caused seawater hypoxia (*36*), which was the main factor limiting the maximum test size of foraminifera (*37*). Another important factor is the synergistic effects from carbon emissions, including global warming and hypoxia (*38*), which are similar to the effects of the large igneous provinces during the F-F, G-L, P-T, T-J, and K-Pg extinction periods. However, extreme environments cannot explain differences in body size selectivity differences in mass extinction events. For example, in the T-J mass extinction event, environmental damage was severe and foraminiferal extinction rates were high (43.02%; table S1), but foraminiferal body size selectivity was not significant (*P* = 1.00).

### The role of size distribution in extinction selectivity

**Fig. 3.**
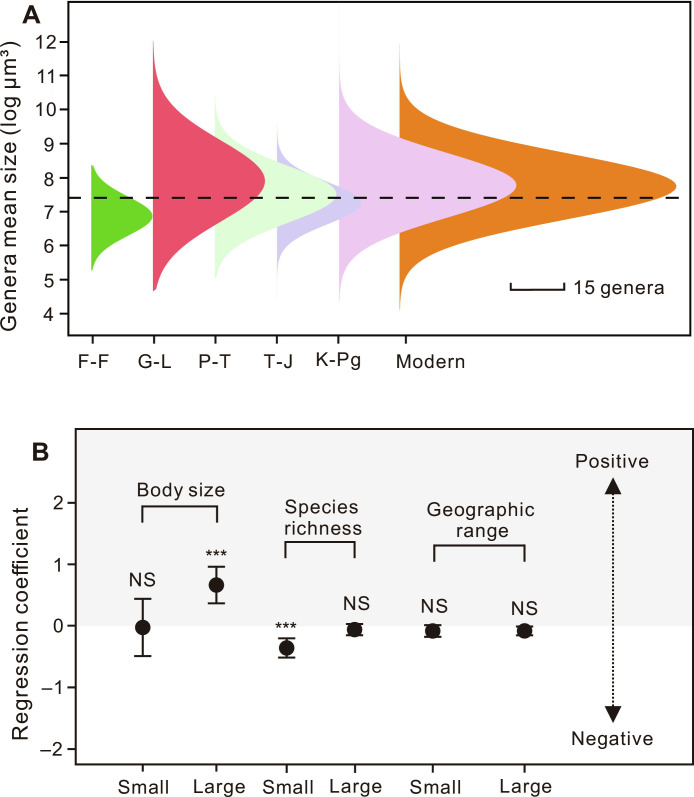
Extinction selectivity and size distribution of foraminifera during mass extinctions. (**A**) Body size distribution of all benthic foraminiferal test volumes (log_10_ μm^3^) during mass extinctions and modern extinction risk. The dashed line indicates the maximum test volume of spherical foraminifera under suboxic conditions (<0.005 mol/m^3^). Abbreviations are consistent with those above. Scale bar, 15 genera. (**B**) Extinction selectivity of the large (≥7.4 log_10_ μm^3^) and small (<7.4 log_10_ μm^3^) foraminiferal groups with respect to body size, species richness, and geographic range. Error bars represent 95% confidence intervals of the estimated correlation coefficients. Gray shading indicates a positive correlation between the variables and extinction risk. ***P < 0.001; NS, *P* > 0.05.

We proposed that body-size distributions may drive differences in extinction selectivity of foraminifera during mass extinctions. For events with significant extinction selectivity, such as the G-L, P-T, and K-Pg extinctions, foraminifera had larger sizes with mean values of 7.91, 7.53, and 7.73 log_10_ μm^3^, respectively (Fig. 3A). In contrast, for events without obvious extinction selectivity, such as the F-F and T-J mass extinctions, foraminifera had smaller sizes with mean values of 6.89 and 7.29 log_10_ μm^3^, respectively. In modern extinction risk, a unique aspect is the larger average size of foraminifera (7.78 log_10_ μm^3^), yet the extinction selectivity shows no statistical significance. (Fig. 3A and fig. S2).

When we combined foraminifera of mass extinctions and modern extinction risk for the analysis, the large group of all foraminiferal genera ≥7.4 log_10_ μm^3^ (based on the maximum sustainable volume of spherical foraminifera in suboxic conditions) showed significant extinction selectivity (Fig. 3B and table S2). In contrast, the small foraminiferal group (<7.4 log_10_ μm^3^) showed no significant body size selectivity. This dependence of extinction selectivity on size distribution is also observed across mass extinctions. Large foraminifera (≥7.4 log_10_ μm^3^) accounted for 62% (85 of 138 = 61.59%), 49% (69 of 142 = 48.59%), and 57% (125 of 218 = 57.33%) in the Capitanian, Changhsingian, and Maastrichtian stages (Fig. 3A and fig. S2). Foraminifera exhibited apparent size selectivity, i.e., large individuals were preferentially extinct during the G-L, P-T, and K-Pg extinction events (Fig. 2A). However, size-related extinction selectivity is not significant in the time bins with lower proportions of large foraminifera (less than 50% genera with body size ≥7.4 log_10_ μm^3^), e.g., F-F (9 of 43 = 21%) and T-J (36 of 86 = 42%). To address the potential impact of sampling bias on our results, we conducted a resampling analysis for the F-F and T-J mass extinction events. Specifically, we resampled the data for these events 1000 times to compare the size differences between extinct and survival genera (data S4). Our results show that there is an 84.4% probability of nonsignificance in the F-F mass extinction. For the T-J mass extinction, there is a 99.3% probability of nonsignificance.

### The potential causes for size distribution–related selectivity

**Fig. 4.**
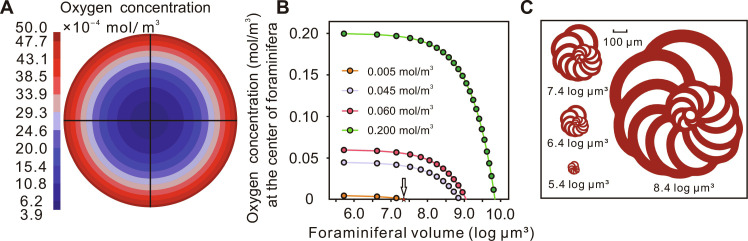
Distribution of oxygen concentration in foraminifera. (**A**) Oxygen distribution in spherical foraminifera (7.4 log_10_ μm^3^) under suboxic conditions, 0.005 mol/m^3^, shown in section. (**B**) Oxygen concentration in the center of spherical foraminifera under four oxygen concentrations simulated by COMSOL (oxygen-rich condition, 0.2 mol/m^3^; hypoxic condition, 0.06 and 0.045 mol/m^3^; suboxic condition, 0.005 mol/m^3^). A solid circle represents the oxygen concentration at the center of foraminifera of different sizes. (**C**) Schematic illustration of foraminifera in various sizes.

Following the previous discussion, these extinction events studied consistently correlate with oceanic anoxia, although the specific degrees of hypoxia vary across each event [(*3*) and fig. S1]. We therefore explored the relationship between ocean deoxygenation and the size distribution of foraminifera. We performed a series of simulations using COMSOL Multiphysics 6.0 software with Fick’s first law as the governing equation, mainly to measure the oxygen content distribution in foraminifera (Fig. 4A). The maximum possible size is limited by the diffusion of oxygen in the organism (*37*). In this study, we quantified the maximum sizes of foraminifera in different oxygen environments by diffusion and consumption of oxygen in foraminifera. In suboxic seawater (0.005 mol/m^3^), 180 μm (7.4 log_10_ μm^3^) is the maximum distance for oxygen diffusion in benthic spherical foraminifera (Fig. 4B). Because of the morphological variations of foraminifera in response to changes in oxygen concentration (*39*, *40*), our study specifically focuses on simulating the spherical foraminifera, as a sphere presents the greatest challenge for oxygen diffusion to the center (*33*). In contrast, other shapes of foraminifera, such as those with flattened sides, may facilitate easier oxygen diffusion, thereby reducing the distance oxygen needs to travel. This value applies to many foraminiferal species because of their sphere-like shape such as ellipsoidal and short columnar. Other shaped foraminifera (e.g., discoid, long columnar) can grow larger at the same oxygen content and diffusion conditions (*41*). The simulation results show that the dependence of the size distribution is controlled by the distance of oxygen diffusion in the foraminifera. The larger the size of the foraminifera, the more difficult it is to get enough oxygen inside. It is also the main driver of the Lilliput effect observed in foraminifera during mass extinction events (*15*–*19*, *42*, *43*).

While hypoxia may well explain the size distribution–dependent selectivity, it does not exclude other causes, such as warming and acidification, which have occurred frequently in past extinction events (*3*, *44*–*46*). Modern experiments show that large foraminiferal individuals are more susceptible to warming and acidification (*47*–*49*). In addition, many large foraminifera live in symbiosis with groups of algae such as diatoms, dinoflagellates, and unicellular chlorophytes and are thus affected by algal extinction (*50*–*52*). For example, the impact event at K-Pg boundary affected algal photosynthesis (*30*, *53*), which would have been an essential contributor to the preferential extinction of large foraminifera.

### Implications for future ocean deoxygenation

Notably, foraminifera larger than 7.4 log_10_ μm^3^ account for more than 50% (267 of 425 = 63%) of genera, yet size selectivity is not significant in modern extinction risk. This phenomenon may be attributed to the following two points. One potential factor is the methodology used in evaluating extinction risk for modern foraminifera. The assessment of modern foraminiferal extinction risk predominantly relies on the application of International Union for Conservation of Nature (IUCN) criteria A and B (*54*, *55*). These criteria consider factors such as a population decline of more than 30% over the past 10 years, a highly fragmented distribution or presence in fewer than 10 locations (fig. S5). This approach applied to modern foraminifera, differs from that used in past extinction events, potentially leading to a methodological bias. Another potential factor for unapparent selectivity in foraminiferal size in modern extinction risk is the small magnitude of temperature rise. Global temperatures are projected to increase by just more than 1°C by 2023 compared to the preindustrial levels (*38*), which is much lower than the magnitudes of temperature change during past global warming events (*44*). However, the selectivity trend for large foraminifera will become more pronounced with increasing human activity, global warming, and ocean deoxygenation. Under a very high emissions scenario (SSP5-8.5), global temperature will increase by 4.7°C by 2100 (*56*), close to the temperature change threshold (5.2°C) for the big five mass extinctions (*22*). Temperature-dependent hypoxia would likely lead to severe extinction and size selectivity of foraminifera.

The present study suggests that protozoa are similar to metazoans in that large species are more likely to be at risk of extinction under future environmental changes. The body size of fish and mammals is also positively associated with extinction risk in modern times (*9*, *12*). However, the factors influencing this size selectivity are different. Large vertebrates, especially fishes, have high nutritional levels and economic value and are therefore more vulnerable to anthropogenic predation (*12*). Foraminifera are strongly influenced by human activities and can be an indicator of marine pollution, although they are not economically important to humans (*35*). Hypoxia limits the maximum size of foraminifera and causes preferential extinction of large foraminifera (Figs. 2 and 4B). Other animals that rely on diffusion for oxygen should also be sensitive to hypoxia. For example, hypoxia caused by human activities also threatens corals with extinction (*57*). This study represents the first assessment of extinction risk for foraminifera not evaluated by the IUCN. In addition, it includes a logistic regression analysis between foraminiferal body size and extinction risk. The findings reveal a consistent pattern in the relationship between foraminiferal body size and extinction risk during extinction events, suggesting that larger foraminifera are more susceptible to extinction. Although the relationship in modern extinction risk is not statistically significant, the parallel extinction trends in foraminifera during both modern times and past extinctions (fig. S1) lead us to infer that benthic foraminifera, especially large-bodied taxa, are likely to experience greater diversity loss in the future than small-sized foraminifera, especially under high and very high emission scenarios (SSP3-7 and SSP5-8.5).

## MATERIALS AND METHODS

### Body size data collection

First, we collected published papers with foraminiferal images from the Frasnian (382.7 to 372.2 Ma), Capitanian (264.28 to 259.51 Ma), Changhsingian (254.14 to 251.902 Ma), Rhaetian (208.5 to 201.3 Ma), and Maastrichtian (72.1 to 66.1 Ma). Second, considering the diverse morphologies of foraminifera (*58*), we adopted test volume as the standard measure of body size. This volume is approximated using various shapes, including spheres, hemispheroids, cones, oval cylinders, oval discoids, cylinders, discoids, and ellipsoids. Third, to quantify the volumes of various foraminiferal morphologies, we used different metrics. For spherical foraminifera, we measured the diameter; however, calculating the volumes of discoidal, conical, and cylindrical foraminifera required measuring both diameter and height. For ellipsoidal tests, we used three diameters, whereas oval discoids, cones, and cylinders require two diameters and a height for accurate test volume calculations. For some specimens, where there is no way to measure the length of both axes, we calculate the length of the unknown axis based on the aspect ratio of the type species. Last, the test volumes were calculated on a logarithmic scale with base 10 due to large individual differences (*59*), and the specific calculation is shown in Feng *et al.* (*15*). A total of 12,701 specimens were included, comprising 381 specimens from the F-F mass extinction event, 1469 specimens from the G-L extinction event, 5501 specimens from the P-T mass extinction event, 1019 specimens from the T-J mass extinction event, 1332 specimens from the K-Pg mass extinction event, and 2993 specimens associated with modern extinction risk. The body size of modern foraminifera is determined by measuring the type specimens.

### Extinction risk data collection

In past extinction events, we defined whether a species was extinct or not by checking whether it crossed the extinction horizon, which is mainly determined according to the Paleobiology Database (https://paleobiodb.org). However, modern foraminiferal species, as well as other microorganisms, have not been included in the IUCN Red List of Threatened Species (*54*)*.* Consequently, the extinction risk classification primarily follows the IUCN Red List criteria A and B. These include a population decline of ≥30% over the past 10 years, highly fragmented distribution or presence in fewer than 10 locations. (*55*). According to the Red List categories, foraminifera are threatened with extinction if they are in the status of “Vulnerable,” “Endangered,” and “Critically Endangered” (*60*). This means that genera experiencing at least a 30% decline in foraminiferal populations over the past 10 years, or those known to exist in no more than 10 locations, are defined as threatened (fig. S5).

### Data reliability analysis

All data were analyzed at the genus level because of its better continuity (*8*), which ameliorated the variability due to the shorter survival time of species. The mean test volume (in log_10_ μm^3^) of each genus was used for statistical analysis here because the distribution of body size of foraminiferal specimens within each genus was normally distributed, and the size variation within the genus was not notable (fig. S3). The species richness and geographic range of each genus were included in our analysis, which were gathered on the basis of our body size database. To estimate the species richness, we tabulated the number of named species per genus for each stage in our collection. Specimens without a species name or classified as indeterminate species (e.g., sp. or spp.) were excluded from the species richness calculation. We used a global equal-area grid as a measure of geographic range (see data S2).

A total of 12,701 specimens of 2604 species belonging to 1053 benthic foraminiferal genera were included in this analysis to examine the influence of body size, species richness, and geographic range on both past and modern extinction risks (data S1 and S2). In this study, only benthic foraminifera were examined, not planktonic foraminifera, because planktonic foraminifera originated in the Jurassic and could not be compared during the pre-Jurassic mass extinctions. This study includes five extinction events, i.e., the F-F, G-L, P-T, T-J, and K-Pg extinctions, with 43, 138, 142, 86, and 218 genera, respectively. Compared to the number of foraminiferal genera collected by Loeblich Jr, and Tappan (*13*) (fig. S4), our data are sufficient. The end-Ordovician mass extinction was excluded from the analyses because the sample size is too small (44 specimens) to draw strong statistical conclusions. We examined extinction selectivity in the G-L extinction because benthic foraminifera were remarkably affected by this crisis, although the G-L extinction is not one of the big five mass extinctions (*20*). The body sizes of 425 modern benthic foraminiferal genera were also included.

### Logistic regression analysis

We used multiple-factor binary logistic regression to assess extinction selectivity of benthic foraminifera under three continuous variables: body size, species richness, and geographic range. Logistic regression is a generalized linear regression analysis model used to solve dichotomous (0 or 1) variables (*61*), such as victim or survivor, in this study. This method has been widely used to estimate the probability of extinction under different explanatory variables, such as geographic range (*6*, *7*) and body size (*5*). The logistic regression analysis in this study was performed using the R 4.3.2 software (*62*). A zero coefficient represented no association; a positive coefficient represented a positive relationship between body size, species richness, geographic range, and extinction risk; and a negative coefficient represented an inverse relationship. We also used the Mann-Whitney *U* test in the R 4.3.2 software to compare the significance of differences between the body sizes of extinct and surviving foraminifera, i.e., the overall median test size of the extinct and surviving genera. Furthermore, we applied a Bonferroni correction to account for multiple comparisons and reduce the chance of false-positive results (*63*). The data and code are available in data S1 to S4.

### Oxygen diffusion model

Oxygen is an essential for the survival of foraminifera and is mainly transported from the environment to within foraminifera by diffusion, a process described by Fick’s first law (*64*). Cytoplasmic flow contributes to oxygen transport as well. In foraminifera, this is especially evident in the pseudopods, where cytoplasmic streaming occurs at flow rates ranging from 0.5 to 7 μm/s (*65*). However, under extreme environmental conditions, the pseudopods of foraminifera retract, leading to a slowdown and eventual cessation of cytoplasmic streaming within approximately 3 min (*66*, *67*). In contrast, diffusion remains largely unaffected by these conditions and continues to occur. Therefore, in extreme environments like hypoxia, diffusion serves as the primary mode of oxygen transport, which matches the observational and modeling data quite well (*64*, *68*). The need for oxygen increases with the sizes of foraminifera, while the supply of oxygen is determined by the concentration in the environment. To determine the relationship between the maximum sizes supported and the ambient oxygen concentration, we used COMSOL Multiphysics 6.0 to determine the maximum sizes of foraminifera that can be supported by a given oxygen concentration. COMSOL Multiphysics 6.0 is an advanced computer-aided engineering software with multiple physical fields, chemical processes, and other simulation capabilities (*69*, *70*). We assumed that the ingress of oxygen molecules into tissues is solely facilitated by diffusion, which can be described mathematically by Fick’s first law (Eq. 1)

$$J=-D\cdot \frac{\text{dC}}{\text{dx}}$$ (1)

where *J* is the flux per unit time, *D* is the diffusion coefficient [1.7 * 10^−9^ m^2^/s (*64*)], and $\frac{\text{dC}}{\text{dx}}$ is the concentration gradient at any boundary.

We configured Fick’s first law as the governing equation and specified various environmental oxygen concentrations: oxygen-rich condition (0.2 mol/m^3^), hypoxic condition (0.06 and 0.045 mol/m^3^), and suboxic condition (0.005 mol/m^3^) (*71*). In addition, we considered the oxygen consumption rates of foraminifera (1.45*10^−3^ mol/m^3^ s), representing the oxygen consumption of benthic foraminifera at 25°C (*72*), and used finite element analysis to determine the distribution of oxygen concentrations within foraminifera cells (Fig. 4A). We stipulated that foraminifera cannot survive in a given oxygen concentration if negative values are observed at any position, thereby establishing the relationship between their maximum sizes and the surrounding oxygen concentrations. Undeniably, our model is a simplification, and many special cases exist in reality. For instance, foraminifera harboring symbiotic algae can produce oxygen via autotrophic photosynthesis, thereby supplying this oxygen to foraminifera (*73*). In addition, some foraminifera use denitrification for respiration in the presence of insufficient oxygen (*74*).
