# Supplementary material for: High extinction risk in large foraminifera during past and future mass extinctions
Source: Sci Adv. 2024 Aug 7;10(32):eadj8223. doi: 10.1126/sciadv.adj8223 (PMC11305383; doi:10.1126/sciadv.adj8223)
Supplement: Supplementary file 1 — Figs. S1 to S5 Tables S1 and S2 Legends for data S1 to S4 [file sciadv.adj8223_sm.pdf]

Science Advances
AAAS

Supplementary Materials for

High extinction risk in large foraminifera during past and future mass extinctions

Yan Feng *et al.*

Corresponding author: Haijun Song, haijunsong@cug.edu.cn

Sci. Adv. **10**, eadj8223 (2024)
DOI: 10.1126/sciadv.adj8223

The PDF file includes:

Figs. S1 to S5
Tables S1 and S2
Legends for data S1 to S4

Other Supplementary Material for this manuscript includes the following:

Data S1 to S4

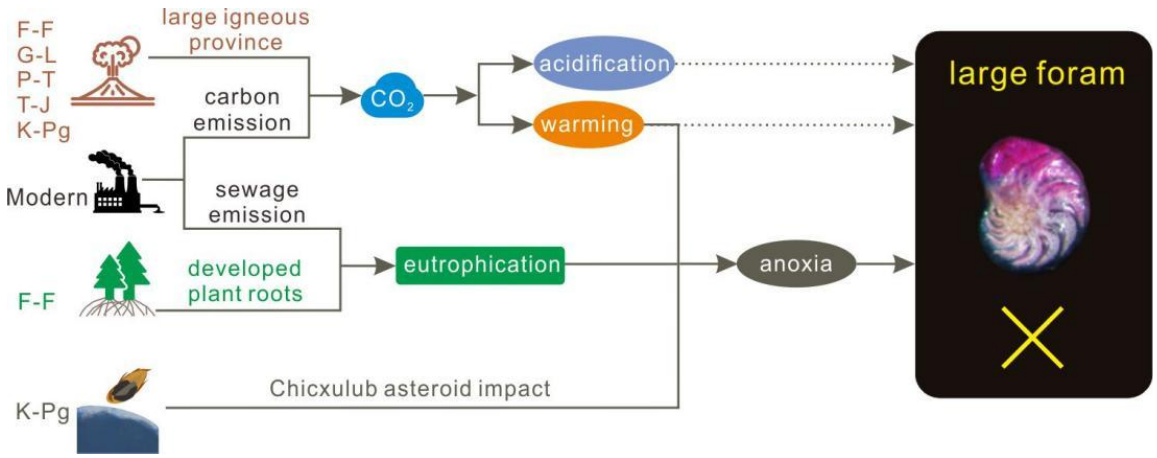

Fig. S1. Causal model of the size-related selectivity of foraminiferal extinction during past extinction events and modern times. F-F, Frasnian-Famennian mass extinction. G-L, Guadalupian-Lopingian extinction. P-T, Permian-Triassic mass extinction. T-J, Triassic-Jurassic mass extinction. K-Pg, Cretaceous-Paleogene mass extinction. Modern, modern era, foram, foraminifera.

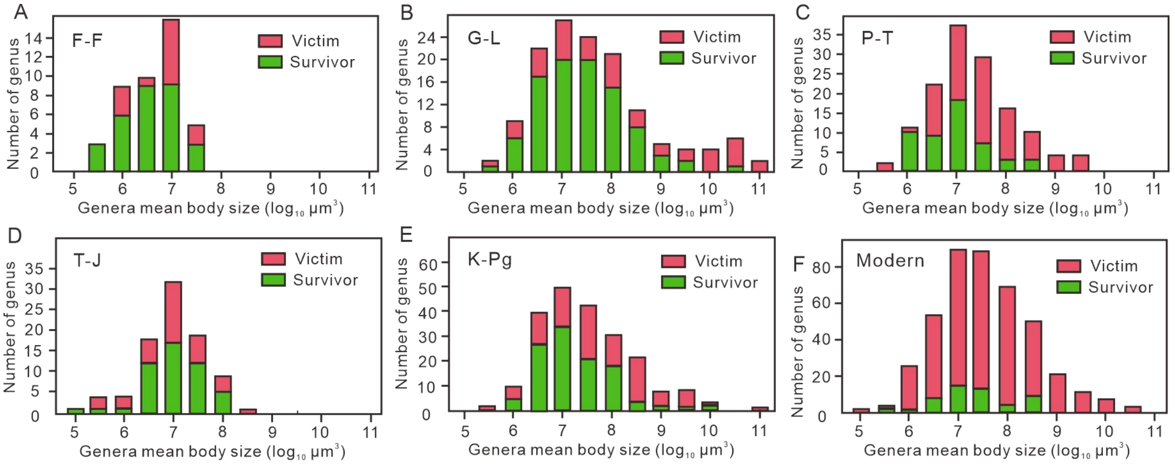

Fig. S2. Histogram of the frequency distribution of benthic foraminiferal test volume ($\log_{10}$ μm^3) during mass extinctions and modern extinction risk. **(A)** Frasnian-Famennian mass extinction (Frasnian). **(B)** Guadalupian-Lopingian extinction (Capitanian). **(C)** Permian-Triassic mass extinction (Changhsingian). **(D)** Triassic-Jurassic mass extinction (Rhaetian). **(E)** Cretaceous-Paleogene mass extinction (Maastrichtian). **(F)** Modern extinction threat. The light green (bottom) and light red (top) shapes represent the distribution of surviving and extinct foraminifera, respectively. The terms "victim" and "survivor" in the modern extinction threat represent foraminiferal taxa that are at extinction risk and those that are not at extinction risk, respectively.

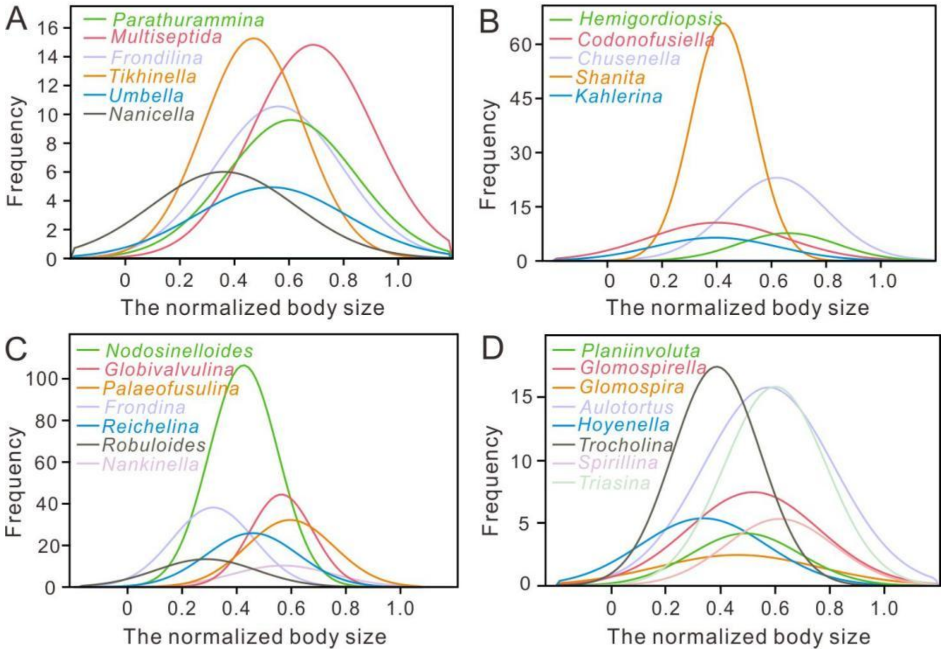

Fig. S3. The normalized body size distribution of benthic foraminiferal genera. (A) Frasnian. **(B)** Capitanian. **(C)** Changhsingian. **(D)** Rhaetian. Because the test volume of each foraminiferal genus varied widely, we mapped the body size of foraminiferal specimens to a range from 0 to 1.

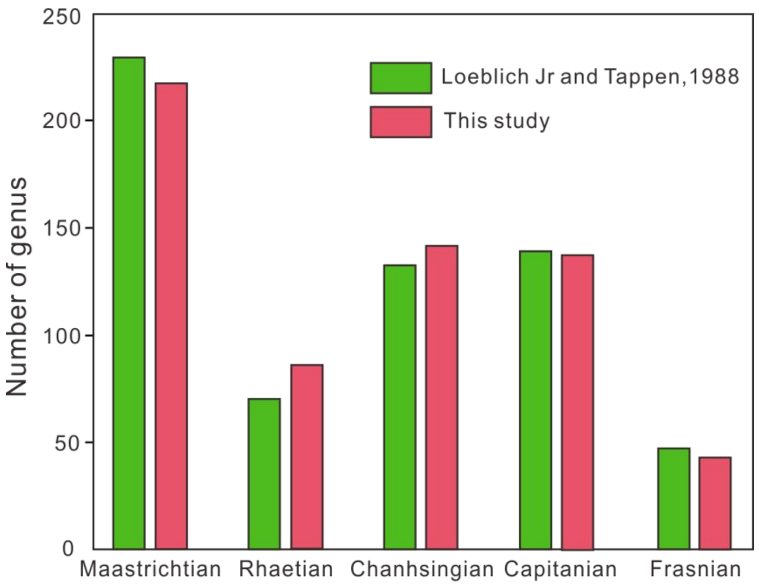

Fig. S4. The number of Maastrichtian, Rhaetian, Changhsingian, Capitanian and Frasnian genera in this study and in Loeblich Jr and Tappan (1988) (*13*).

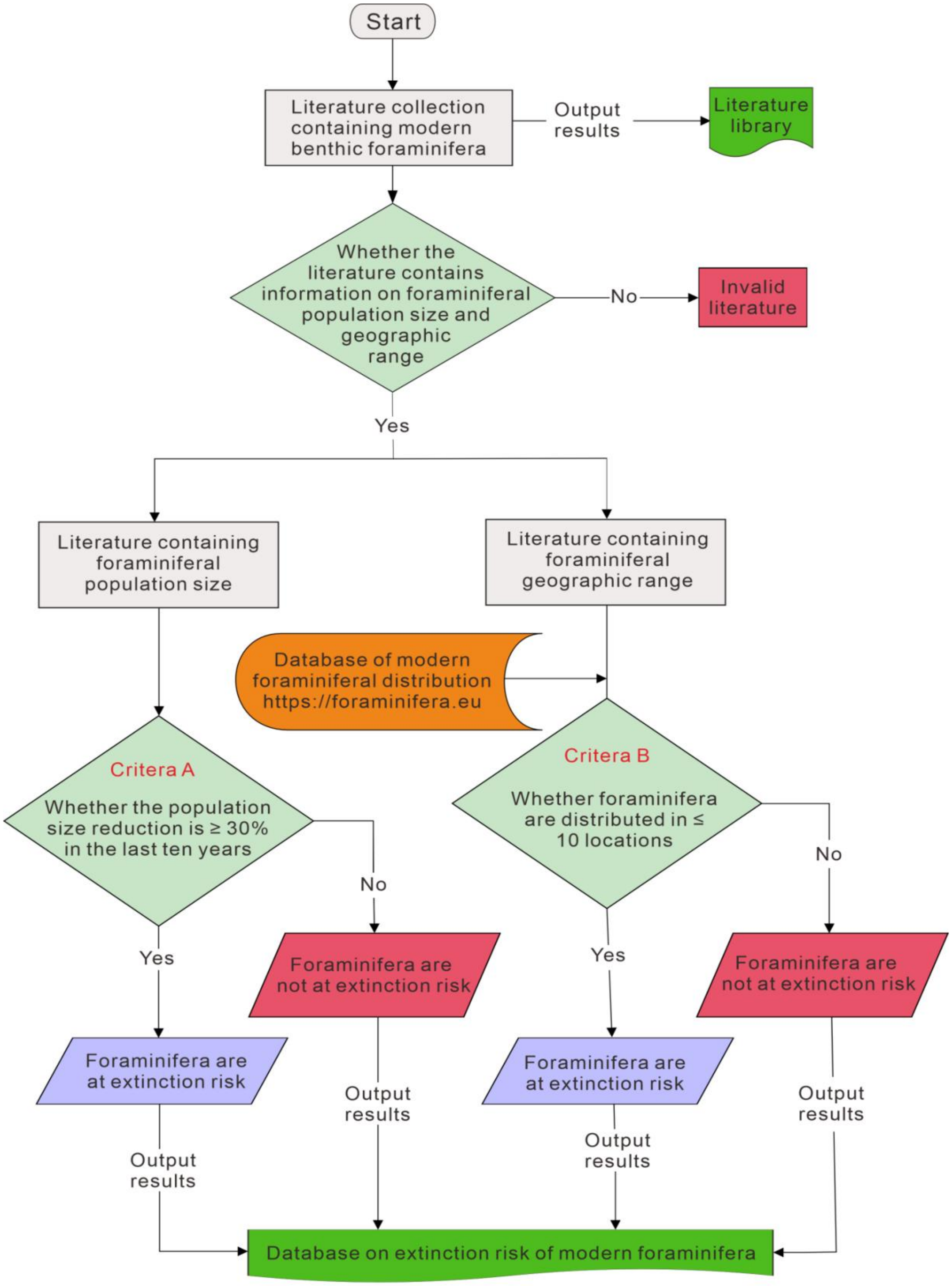

Fig. S5. Flowchart for modern foraminiferal risk assessment. The risk is mainly classified according to *IUCN Red List* criterion A, B (*54, 55*).

Table S1. Medium, maximum, and minimum values ($\log_{10}$ μm^3), Mann-Whitney U tests and Bonferroni corrections for survival and extinct benthic foraminiferal genera in past extinctions and modern extinction risk. N, number of genera. F-F, Frasnian-Famennian mass extinction; G-L, Guadalupian-Lopingian extinction; P-T, Permian-Triassic mass extinction; T-J, Triassic-Jurassic mass extinction; K-Pg, Cretaceous-Paleogene mass extinction. Modern, modern extinction threat. To address the potential impact of sampling bias on our results, we conducted a resampling analysis for the F-F and T-J mass extinction events. Specifically, we resampled the data for these events 1,000 times to compare the size differences between extinct and survival genera (Data S4).

		All genera	Survival genera	Extinct genera	Extinction
F-F	N	43	30	13	30.23%
	Medium volume	6.94	6.68	7.31	
	Maximum volume	7.95	7.72	7.95	
	Minimum volume	5.75	5.75	6.16	
	U test (*p* value)		0.1044		
	Bonferroni correction (*p* value)		0.6266		
G-L	N	138	94	44	31.88%
	Medium volume	7.74	7.67	8.14	
	Maximum volume	11.01	10.55	11.01	
	Minimum volume	5.71	5.99	5.71	
	U test (*p* value)		0.0167		
	Bonferroni correction (*p* value)		0.1006		
P-T	N	142	54	88	61.97%
	Medium volume	7.39	7.15	7.69	
	Maximum volume	9.69	8.87	9.69	
	Minimum volume	5.85	6.17	5.85	
	U test (*p* value)		$3.036*10^{-5}$		
	Bonferroni correction (*p* value)		$1.822*10^{-4}$		
T-J	N	86	49	37	43.02%
	Medium volume	7.32	7.34	7.31	
	Maximum volume	8.85	8.42	8.85	
	Minimum volume	5.22	5.22	5.71	
	U test (*p* value)		0.9309		
	Bonferroni correction (*p* value)		1.00		
K-Pg	N	218	113	105	48.17%

	Medium volume	7.61	7.35	7.94
	Maximum volume	11.94	10.12	11.94
	Minimum volume	5.53	6.24	5.53
	U test (*p* value)		$1.665*10^{-4}$	
	Bonferroni correction (*p* value)		$9.9916*10^{-4}$	
Modern	N	425	46	379
	Medium volume	7.70	7.34	7.74
	Maximum volume	10.80	8.68	10.80
	Minimum volume	5.29	5.64	5.29
	U test (*p* value)		$2.432*10^{-3}$	
	Bonferroni correction (*p* value)		0.01459	

Table S2. Multiple logistic regression analysis of extinction risk with respect to body size, species richness, and geographic range for foraminifera in the F-F, G-L, P-T, T-J, and K-Pg extinctions, and modern extinction risk. Small Group (< 7.4 $\log_{10}$ μm^3) and large group (≥ 7.4 $\log_{10}$ μm^3).

Extinction event	Factor	Regression coefficient	95% confidence interval	*p* value	Bonferroni correction
F-F	Body Size	1.245	1.337	0.068	0.544
	Richness	0.096	0.271	0.505	1.000
	Range	-0.151	0.457	0.530	1.000
G-L	Body Size	0.577	0.339	$8.540*10^{-4}$	$6.832*10^{-3}$
	Richness	0.129	0.285	0.374	1.000
	Range	-0.372	0.369	0.049	0.392
P-T	Body Size	1.103	0.560	$1.140*10^{-4}$	$9.120*10^{-4}$
	Richness	$-2.659*10^{-4}$	0.137	0.997	1.000
	Range	-0.292	0.241	0.018	0.144
T-J	Body Size	0.117	0.745	0.758	1.000
	Richness	-0.422	0.375	0.027	0.216
	Range	0.161	0.389	0.417	1.000
K-Pg	Body Size	0.630	0.320	$1.170*10^{-4}$	$9.36*10^{-4}$
	Richness	-0.259	0.221	0.021	0.168
	Range	-0.130	0.445	0.566	1.000
Modern	Body size	0.303	0.574	0.300	1.000
	Richness	-0.808	0.226	$2.38*10^{-12}$	$1.904*10^{-11}$
Small Group < 7.4 $\log_{10}$ μm^3	Body Size	-0.030	0.467	0.900	1.000
	Richness	-0.364	0.157	$5.560*10^{-6}$	$4.448*10^{-5}$
	Range	-0.087	0.095	0.071	0.568
Large Group > 7.4 $\log_{10}$ μm^3	Body Size	0.662	0.298	$1.350*10^{-5}$	$1.08*10^{-4}$
	Richness	-0.065	0.092	0.170	1.000
	Range	-0.086	0.072	0.020	0.16

Data S1. Raw data of benthic foraminiferal specimens during the past extinction events and modern extinction risk, including body size, geographic distribution, sample number, lithology, environment, and publication reference.

Data S2. Assessment of the species richness and geographic range of benthic foraminiferal genera during past extinctions and modern extinction risk. Body size is the mean of all specimens in each genus. Species richness is the number of named species per genus at each stage. Species without a species name or species designated indeterminate (e.g., sp. or spp.) are not counted. Geographic range is the number of equal geographic grid intervals in which each genus occurs at each stage. The "1" in the "Extinction" column indicates that the foraminifera became extinct during the mass extinctions, while the "0" indicates that the foraminifera survived the mass extinctions. Foraminiferal body size, species richness, and geographic distribution were gathered based on our body size database Data S1. Foraminiferal extinction data during past extinctions were taken from the Paleobiology Database (PBDB) (https://paleobiodb.org). The modern extinction risk is mainly classified according to *IUCN Red List* criterion A and B, namely, population reduction and extent of occurrence (*60*) (fig. S5). F-F, Frasnian-Famennian mass extinction; G-L, Guadalupian-Lopingian extinction; P-T, Permian-Triassic mass extinction; T-J, Triassic-Jurassic mass extinction; K-Pg, Cretaceous-Paleogene mass extinction.

Data S3. Code used for data analysis in this article. All analyses were conducted in R 4.3.2 (*62*).

Data S4. Mann-Whitney U test and Bonferroni correction results for size differences between extinct and survival genera in the Frasnian-Famennian (F-F) and Triassic-Jurassic (T-J) mass extinction events based on 1000 bootstrap resampling iterations.
